# No Banquet

**Published:** 1887-04

**Authors:** 


### NO BANQUET.

In our last we had an article "Banquet or No Banquet," in which it was pointed out that there is a growing opposition on the part of the members to this yearly spread of a "feast and flow."

It is quite natural that each town that is honored by a meeting of the State Medical Association should want to emulate the example of its predecessor, — and the cost of doing so — doubtless, has deterred many smaller towns from inviting such meeting. It was also pointed out that just at this time when the temperance question is being agitated — a prohibition political party formed, and an anti-prohibition plank has been introduced into the platform of the great Democratic party by a few self constituted "leaders" — it was believed that whatever action was had on the subject of entertaining five hundred medical delegates to a State Convention with a wine supper, would be construed into an expression for, or against temperance, and a political significance given it; for those, and other reasons, this Journal opposed the proposal to follow suit — and give our guest a banquet. There are other and powerful reasons against the custom, not yet stated.

The committee of arrangements — the whole resident profession of Austin, at a recent meeting reconsidered the question, and by a vote of 15 to 8 — five absent and not voting — decided to institute an innovation — a new departure — by dispensing with that feature in the proposed entertainment. The wine banquet was voted down by nearly a ⅔ vote.

We are proud of this action. We believe now is the time, and the Capital of the State the place, to put a stop to a custom that is burdensome — at least, and worse than useless — not to say of questionable propriety. No smaller town could have done so without being open to the charge of parsimony. We believe that Austin alone could rise to the dignity of taking the initiative in dispensing with a custom more honored — we say — in the breach than in the observance.

With ample funds in hand it cannot be said that any mercenary motive entered into a consideration of the question.

This action by our committee may give rise to some little dissatisfaction, but we venture the more thoughtful members will heartily applaud it. Perhaps as expensive, and, we think, more elegant, entertainment has been provided in its stead.

"Put not temptation in the way of youth" — nor we say — in the way of the weak, for all men are not strong. Suppose some lately reformed dipsomaniac — reformed after a desperate struggle with the demon, — saved by moral suasion and medical treatment, were present at some wine feast — and — being tempted, should fall? Where would the responsibility rest? Precisely such things have occurred. But it is not from fear that our guests will make beasts of themselves — but from a principle — arising from a sincere regard for the well being of mankind, for the rising and future generations, that the arrangement committee decide not to endorse, by their action, the use of intoxicating liquor; and have thus thrown, — it may be said, — the moral influence of the medical profession in the scale of temperance.

If the voice of the JOURNAL has had any influence in bringing about this decision, we have not labored in vain. Yet, we do not wish to be misunderstood. If there are those with us on that occasion who want wine or liquor, there are, unfortunately, places where it can be had. The committee simply do not propose to set it out; recognizing at the same time the duty of host to guest. They propose to entertain their distinguished guests in an appropriate and, they hope, an acceptable manner; and the action is parrallel, of a temperance man in his own home — who has a distinguished guest whom he desires to honor; he would not depart from his principle, and the rule of his house, and set out wine — under any circumstances! Not that he fears his guest is weak, and might be indiscreet, — but upon principle! Yet — old members may remember *one* banquet where a humiliating occurrence took place; a recurrence of it would simply be disgraceful. The days of wine banquets at medical conventions in Texas, we trust, are over, never to return. *So mote it be.*

It is gratifying to receive, as we have done, numerous letters from distinguished members — endorsing the JOURNAL article referred to.

